# Gestational Trophoblastic Disease: A Multimodality Imaging Approach with Impact on Diagnosis and Management

**DOI:** 10.1155/2014/842751

**Published:** 2014-07-13

**Authors:** Sunita Dhanda, Subhash Ramani, Meenkashi Thakur

**Affiliations:** ^1^Department of Diagnostic Imaging, National University Hospital, Level 2, Main Building, 5 Lower Kent Ridge Road, Singapore 119074; ^2^Tata Memorial Hospital, Dr. E. Borges Marg, Parel, Mumbai, Maharashtra 400012, India

## Abstract

Gestational trophoblastic disease is a condition of uncertain etiology, comprised of hydatiform mole (complete and partial), invasive mole, choriocarcinoma, and placental site trophoblastic tumor. It arises from abnormal proliferation of trophoblastic tissue. Early diagnosis of gestational trophoblastic disease and its potential complications is important for timely and successful management of the condition with preservation of fertility. Initial diagnosis is based on a multimodality approach: encompassing clinical features, serial quantitative *β*-hCG titers, and pelvic ultrasonography. Pelvic magnetic resonance imaging (MRI) is sometimes used as a problem-solving tool to assess the depth of myometrial invasion and extrauterine disease spread in equivocal and complicated cases. Chest radiography, body computed tomography (CT), and brain MRI have been recommended as investigative tools for overall disease staging. Angiography has a role in management of disease complications and metastases. Efficacy of PET (positron emission tomography) and PET/CT in the evaluation of recurrent or metastatic disease has not been adequately investigated yet. This paper discusses the imaging features of gestational trophoblastic disease on various imaging modalities and the role of different imaging techniques in the diagnosis and management of this entity.

## 1. Introduction

Gestational trophoblastic disease (GTD) refers to an abnormal trophoblastic proliferation composed of a broad spectrum of lesions ranging from benign, albeit premalignant hydatiform mole (complete and partial), through to the aggressive invasive mole, choriocarcinoma, and placental site trophoblastic tumor (PSTT). Gestational trophoblastic neoplasia (GTN) refers to the aggressive subset that has a capability for independent growth and metastases and requires chemotherapy. It includes invasive mole, choriocarcinoma, and PSTT. GTN may arise following evacuation of a molar pregnancy as well as after a normal term or preterm pregnancy, abortion, or ectopic pregnancy. Hence, it is also referred to as persistent trophoblastic neoplasia (PTN). These lesions vary considerably in clinicopathologic behavior and propensity for local invasion and metastases. Although GTD may occur as a pregnancy complication in women of any age, it is more common at teenage or advanced maternal age (40–50 years) [1, 2]. Early detection of GTN is important as it has an excellent prognosis following treatment due to exquisite chemosensitivity of most of these lesions [1, 2]. In this paper, we describe the role of various imaging techniques in the diagnosis and management of GTD. A brief overview of the underlying pathophysiology, clinical features, classification, and posttreatment surveillance of the disease is also provided.

## 2. Pathophysiology

Trophoblast is a gestational tissue which covers the blastocyst and provides route for nourishment between the maternal endometrium and the developing embryo in early pregnancy. Ultimately, it covers the surface of chorionic villi and forms the fetal portion of the placenta. Trophoblast is comprised of cytotrophoblast, syncytiotrophoblast, and intermediate trophoblast. Cytotrophoblast shows high mitotic activity; however, it lacks hormone synthesis. Syncytiotrophoblast forms the chorionic villi, has low mitotic activity, and synthesizes beta-human chorionic gonadotrophin (*β*-hCG), which is used as a tumor marker. Intermediate trophoblast has features of the other two components and is responsible for endometrial invasion and implantation. In the various forms of GTD, different components of trophoblast show abnormal proliferation to a variable extent [1]. Although most GTNs secrete *β*-hCG hormone with abnormal elevation of *β*-hCG titers being one of their diagnostic features of GTD, the titers vary in different tumor types. Some choriocarcinomas and bimorphic tumor types secrete only low levels of *β*-hCG [3]. PSTT represents a neoplastic proliferation of intermediate trophoblasts [2]. Unlike other forms of GTD, it is characterized by low *β*-hCG levels due to lack of syncytiotrophoblastic proliferation [2–4]. However, it shows increased expression of tissue as well as serum human placental lactogen (hPL) [4].

Hydatiform mole results from an aberrant fertilization process. It includes complete and partial mole, the former being more common. Complete hydatiform mole arises when a single haploid sperm (23X) fertilizes a chromosomally empty ovum followed by chromosomal duplication and formation of a zygote with no maternal contribution. This leads to a genetically diploid pattern (more commonly 46XX) in a complete mole. Similar fertilization and duplication with a 23Y sperm do not produce a viable (46YY) zygote. Rarely, 46XY pattern may result due to fertilization of a chromosomally empty ovum by two different sperms. In contrast, partial hydatiform mole usually has a triploid chromosomal pattern (46XXX, 46XXY, and 46XYY), resulting from fertilization of a normal egg by two sperms [1, 2, 5, 6].

Pathologically, hydatiform mole is composed of abnormally proliferating syncytiotrophoblastic and cytotrophoblastic cells resulting in generalized swelling of chorionic villi. Complete mole shows severe villous swelling, resembling a bunch of grapes and usually an absence of embryo. Villous swelling is less intense in a partial mole and an embryo is usually present. The embryo may survive up to early second trimester [1, 2]. Complete or partial hydatiform mole invading the myometrium is called invasive mole. Hydatiform mole is a premalignant condition with 16% of complete and 0.5% partial moles undergoing transformation into malignant forms (invasive mole, choriocarcinoma, or PSTT) [1, 2]. Choriocarcinoma consists of malignant proliferation of cytotrophoblast and syncytiotrophoblast in various proportions; however, it is histopathologically distinct from invasive mole in that it lacks chorionic villous formations. It may arise following hydatiform mole, term pregnancy, miscarriage, or ectopic pregnancy. Choriocarcinoma is a highly malignant, necrotic, hemorrhagic, and locally invasive form of GTN. Early and extensive vascular invasion results in metastases even when the primary tumor is quite small [1, 2]. Choriocarcinoma arising after miscarriage or term pregnancy may present after several years, directly as metastases, elevated *β*-hCG levels, and normal pelvic sonography findings [2, 6]. PSTT is the rarest form of GTN with uncertain biological behavior [2, 4, 7]. It arises from the placental implantation site following a normal pregnancy, abortion, or hydatiform mole, most commonly from an antecedent normal pregnancy. It is generally a slow growing tumor with a tendency for local and lymph nodal metastases before distant metastases (a very rare feature in choriocarcinoma) [2].

## 3. Clinical and Laboratory Features

Initial diagnosis of GTD is based on a combination of history, examination, quantitative *β*-hCG titers, and pelvic sonography. Diagnosis of hydatiform mole based on clinical features may be difficult due to nonspecificity of signs and symptoms. Patients present with irregular vaginal bleeding, excessive vomiting, transvaginal expulsion of grape-like vesicles, abnormally enlarged uterus, and features of preeclampsia, anemia, or hyperthyroidism [1, 5, 8]. *β*-hCG levels show large variation in normal, multiple, and abnormal gestations and when considered in isolation may be misleading for diagnosis of hydatiform mole. Hence, early first trimester sonography remains the investigation of choice for initial diagnosis of hydatiform mole [1, 8].

The standard treatment for hydatiform form is suction evacuation, resulting in approximately 84% cure rate for complete moles and 99.5% for partial moles [1, 2]. It is difficult to predict patient outcome at the time of initial diagnosis. Hence, all patients are followed up with serial quantitative serum *β*-hCG measurements following suction evacuation, to allow early diagnosis of persistent trophoblastic neoplasia [1, 5, 9]. *β*-hCG levels should decline following suction evacuation. As per the latest FIGO guidelines [10], posthydatiform mole GTN may be diagnosed based on any of the following criteria:

1. *β*-hCG level plateau for 4 measurements over a period of 3 weeks or longer, that is, for days 1, 7, 14, and 21;
2. a rise in *β*-hCG levels for 3 consecutive measurements or longer over a period of at least 2 weeks or more, that is, on days 1, 7, and 14;
3. histological diagnosis of choriocarcinoma;
4. elevated *β*-hCG levels for 6 months or more after evacuation.

Although *β*-hCG is useful for diagnosis of PTN, imaging studies may play a confirmatory role in early disease or patients with confusing clinical picture. The most important role of ultrasound in patients with suspected PTN is to exclude a normal gestation as a cause of elevated *β*-hCG level [1].

Nonmetastatic GTN presents as a localized uterine tumor with abnormal vaginal bleeding. Uterine perforation may result in intraperitoneal bleed, presenting as an acute abdomen. Metastases have been reported in 19% of cases, most commonly from choriocarcinoma [2]. Hematogenous dissemination is the principal route of spread, most commonly to lung (80%) followed by vagina (30%), brain (10%), and liver (10%). It may also metastasize to kidney, gastrointestinal tract, skin, or fetus (from choriocarcinoma). Isolated metastasis to other sites is rare in the absence of lung and vaginal metastases [2, 6, 9]. Hypervascular metastases may present with features of hemorrhage. Lung metastases may be asymptomatic or present with hemoptysis, dyspnea, chest pain, or pulmonary artery hypertension. Vaginal metastases may result in torrential vaginal bleeding. Hence, a biopsy should be avoided. Brain metastases may present with headache, seizures, motor, or sensory deficit. Liver metastases are usually asymptomatic [2, 9].

## 4. Imaging Features of the Uterine Disease

### 4.1. Ultrasound

Grey-scale ultrasound with color and spectral Doppler imaging is a very useful tool for diagnosing GTD, determining presence of invasive disease, predicting response to chemotherapy, postchemotherapy follow-up, and detection of recurrence [2, 6].

#### 4.1.1. Ultrasonographic Features of Hydatiform Mole

**Figure 1:**
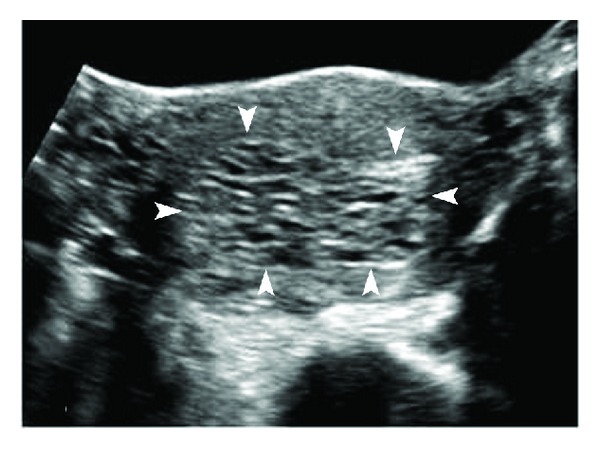
Complete hydatiform mole. Transverse transabdominal sonography (TAS) image of the uterus shows distension of the uterine cavity by echogenic material with numerous small, irregular cystic spaces within (arrowheads). The normal hypoechoic myometrium can be seen stretched at the periphery. There is no identifiable fetal tissue.

Ultrasound is the first line imaging investigation for diagnosis of a clinically suspected hydatiform mole since a single abnormally elevated serum *β*-hCG level measured at the time of patient presentation is not diagnostic and may be seen in a multiple gestation as well [2, 6, 8, 11, 12]. Pelvic sonography is performed as a routine investigation during early pregnancy to accurately date the gestation and determine any abnormalities [6, 11]. Ultrasound can be done by transabdominal or transvaginal approach. Transvaginal sonography provides better details of the lesion due to its superior spatial resolution and proximity to the anatomy of interest. On the contrary, transabdominal approach needs the patient to hold urine resulting in patient discomfort and provides fewer details. On first trimester transabdominal ultrasound, hydatiform mole which constitutes 80% cases of GTD will be seen most frequently as an enlarged uterus with a heterogeneous endometrial mass of variable echogenicity (predominantly echogenic) [1, 2, 6, 12]. Ultrasound appearance of the lesion has been classically described earlier as “snowstorm” or “granular” due to multiple echogenic foci [1]. Transvaginal sonography (TVS) can better demonstrate the lesion morphology and any myometrial invasion. Fluid-filled molar vesicles, representing hydropic, and swollen villi are typically seen as multiple small anechoic spaces varying in size from 1–30 mm throughout the lesion on first trimester transvaginal sonography (Figure 1). With increasing gestational age, the anechoic spaces become larger and more numerous due to presence of prominent villi, making sonographic diagnosis of hydatiform mole easier in second trimester than in the first trimester even by transabdominal approach [2, 6]. Uterine volume should also be accurately estimated on ultrasound as it correlates with the tumor burden and hence determines risk stratification [2].

A fetus or fetal parts are not seen in a complete hydatiform mole except in 1-2% of cases with coexistent dizygotic diploid twin pregnancy [1]. In contrast, partial mole is usually associated with a fetus which is growth retarded or anomalous and an enlarged, thickened placenta with numerous anechoic cystic lesions. A dizygotic diploid twin pregnancy coexistent with a complete mole can be differentiated from a triploid partial mole on ultrasound by identifying a separate normal placenta in the former [1, 2, 6]. Placental appearance similar to partial mole may result from hydropic placental degeneration associated with first trimester embryonic demise of any cause [2, 6].

Differentiation of the complete and partial moles can be difficult but is of limited clinical significance, as the management is similar [6].

**Figure 2:**
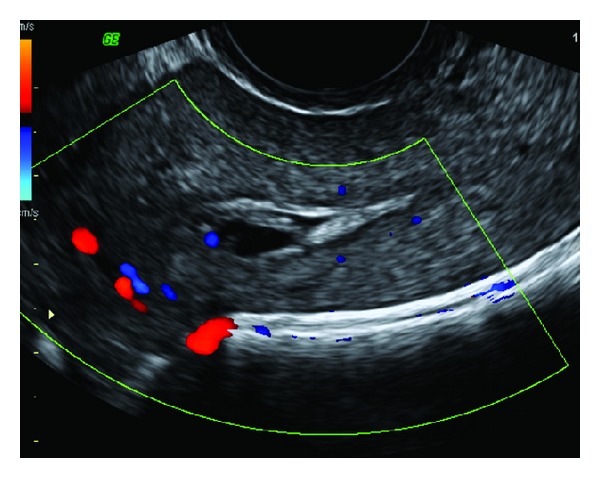
Complete mole in a patient with 8-week amenorrhoea resembling a blighted ovum. Sagittal transvaginal sonography (TVS) shows an anechoic empty gestational sac-like structure with thin echogenic lining and a size smaller than expected for the gestational age.

Although ultrasound is very useful for suggesting a molar pregnancy, final diagnosis still rests with the pathology [2]. Ultrasound finding of a heterogeneous endometrial mass is nonspecific and may also be seen in retained products of conception [2, 8]. Sometimes, it may appear as a large, central fluid collection, mimicking an anembryonic gestation or miscarriage (Figure 2) [1]. However, correlation with clinical features and *β*-hCG will be useful in making the distinction.

Ovaries may show theca lutein cysts due to hyperstimulation by high circulating gonadotropin levels in up to 40% of cases. The cysts are multilocular and usually bilateral. Rarely, these may hemorrhage or rupture leading to an acute abdomen. They usually resolve within a few months after treatment of the intrauterine process [1].

#### 4.1.2. Ultrasonographic Features of Invasive Disease (GTN)

**Figure 3:**
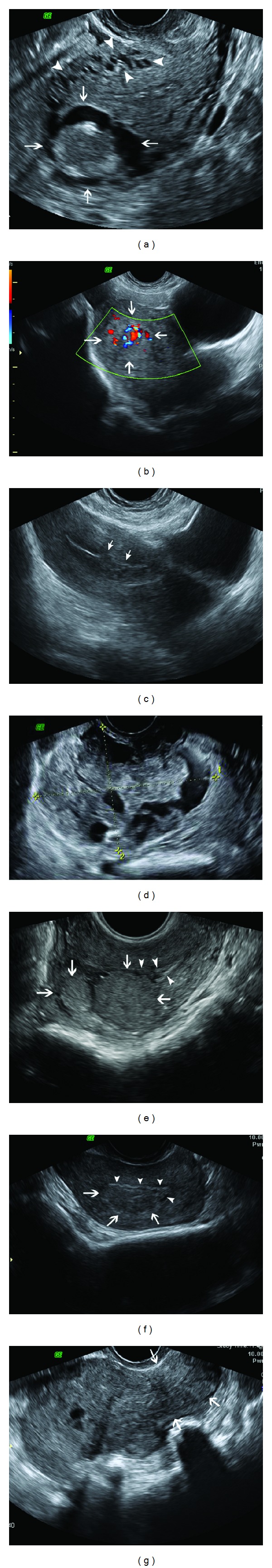
Sonographic spectrum of GTN. (a) Complex solid-cystic mass in the uterine fundus with myometrial epicenter (arrows) on sagittal TVS. Anechoic serpiginous structures in the adjoining myometrium represent increased vascularity (arrowheads). (b) Predominantly echogenic myometrial mass (arrows) effacing the zonal anatomy of uterus on Sagittal TAS. Color flow Doppler ultrasound reveals prominent vascularity in the lesion. (c) Small hypoechoic lesion (arrows) with irregular margins in the lower uterine body, disrupting the endometrial stripe and invading the adjoining myometrium on Sagittal TAS. (d) Ill-defined multicystic myometrial mass (between cursors) on transverse TVS. (e) Two round well-defined submucosal myometrial GTN lesions (arrows) with homogeneous isoechoic to hyperechoic echotexture, indenting the endometrial stripe (arrowheads) and partially effacing it on sagittal TVS, resembling submucosal fibroids. (f) An ovoid isoechoic mass with irregular margins (arrows) at endomyometrial interface on sagittal TVS simulating an adenomyoma or a submucosal fibroid. Arrowheads = endometrium (g) Enlarged uterus with a lobulated contour and heterogeneous echotexture on sagittal TVS. Arrows = cervix.

Myometrial invasion is best appreciated on TVS due to superior demonstration of the interface between trophoblastic tissue and myometrium [2]. Invasive mole, choriocarcinoma, and PSTT are seen on grey-scale ultrasound as nonspecific focal masses (Figures 3(a)–3(f)) with myometrial epicenter and are sonographically indistinguishable from one another. The mass may be echogenic, hypoechoic, complex, or multicystic. It may show anechoic spaces which represent hemorrhage, necrosis, cysts, or vascular spaces. More extensive disease may appear as a heterogeneously enlarged uterus with lobulated contour or large pelvic mass which may extend to involve other pelvic organs (Figure 3(g)) [12]. These masses may be potentially confused with fibroids or adenomyosis. Adenomyosis typically appears as a diffuse disease process causing enlarged uterus with a diffuse heterogeneous echo texture. It can also manifest as asymmetrical myometrial thickening, myometrial cysts, indistinct endometrial-myometrial junction, polyploid lesion, or focal mass within the myometrium with poorly defined margins that blend with the surrounding myometrium. Typical fibroid usually presents as a well-circumscribed hypoechoic myometrial lesion, although echogenicity may vary. The various ultrasound presentations of gestational trophoblastic neoplasia may overlap with these imaging findings of fibroids and adenomyosis. However, correlation with serum *β*-hCG levels, clinical history, and lack of extreme vascularity on Doppler sonography aid in their differentiation [12, 13].

Persistent trophoblastic neoplasia is presumed to be invasive mole unless there is presence of metastases to suggest choriocarcinoma. Since both invasive mole and choriocarcinoma are usually treated with chemotherapy, histological differentiation of the two types is not needed routinely [1]. With effective chemotherapy, the lesions usually become progressively smaller and hypoechoic on ultrasound [12]. However, differentiation of PSTT from invasive mole or choriocarcinoma is important because it is relatively chemoresistant and often requires hysterectomy for treatment [2, 6]. Although invasive mole, choriocarcinoma and PSTT are indistinguishable sonographically, the diagnosis of PSTT is strongly suggested when there are sonographic features of GTN with very low levels of *β*-hCG [6].

#### 4.1.3. Role of Doppler Imaging

**Figure 4:**
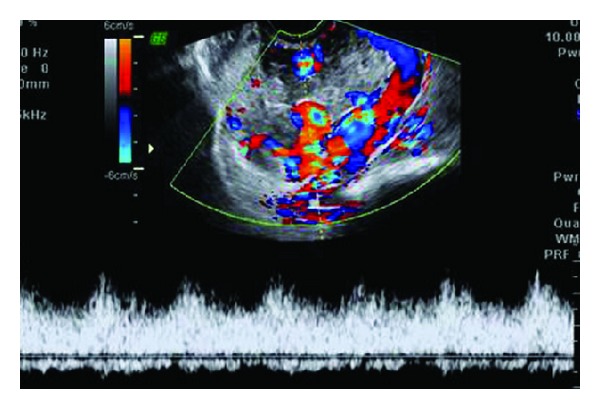
Color flow Doppler of the same patient as in Figure 3(d) reveals a mosaic pattern of color signal within the cystic spaces representing turbulent flow. Spectral analysis of the abnormal vasculature reveals high velocity low impedance pulsatile flow.

Color flow and spectral Doppler are routinely performed in addition to grey-scale ultrasound for diagnosis of the primary or recurrent GTD and posttreatment follow-up. In a normal pregnancy, first trimester Doppler study of the intrauterine arteries shows high resistance flow with low diastolic velocities except at the implantation site [2, 6]. The flow resistance reduces in the second and third trimesters with increasing physiological arterial invasion by trophoblastic tissue. In contrast, molar pregnancy shows high velocity, low impedance waveforms in the first and early second trimesters themselves due to high degree of arterial invasion by abnormally proliferating trophoblast. Arterio-venous shunts associated with neovascularization within the invasive myometrial mass result in an appearance of chaotic vasculature with color aliasing and loss of vascular discreteness on Color Doppler imaging (Figure 4). This extreme vascularity appears as high velocity and low impedance flow [2, 6, 12]. Vascular impedance can be quantified using indices derived from the uterine artery waveform known as pulsatility index (PI) and resistive index (RI). High resistance flow produces high PI and RI, and vice versa. Although there are no unanimously agreed cut-off values for these indices, an RI of less than 0.4 and a PI of less than 1.5 indicative of low uterine artery resistance have been observed in gestational trophoblastic neoplasia [2, 14, 15]. Doppler ultrasound can help determine presence of invasive disease by demonstrating extension of this abnormal vascularity into the myometrium [6].

These Doppler ultrasound features may also be seen due to any cause of increased pelvic blood flow such as retained products of conception, ectopic pregnancy, pelvic inflammation, nontrophoblastic pelvic malignancy, or uterine arterio-venous malformation [12]. Zhou et al. [14] observed lower resistive indices (RI) in invasive mole and choriocarcinoma than complete or partial hydatiform mole indicative of greater degree of vascular invasion in the first two.

PSTT may be hypovascular or hypervascular, with the former form being not associated with prominent vascularity on Doppler ultrasound [2, 12].

Studies by Long et al. [16] and Agarwal et al. [15] have suggested PI to be a potential surrogate marker to predict response of GTN to chemotherapy. Patients with low pulsatility index (PI) have been shown to be more likely to become chemoresistant to single drug therapy with methotrexate.

Recently published studies by Agarwal et al. [17] and Sita-Lumsden [18] have revalidated the usefulness of uterine artery PI (UAPI) as a predictor of methotrexate resistance (MTX-R) independent of Charing Cross Hospital and FIGO scores. The risk of MTX-R in patients with a FIGO score of 6 and UAPI was shown to be 100% versus 20% in patients with PI > 1 (*χ* ^2^ *P* < 0.0001) by Agarwal et al. Sita-Lumsden et al. showed that UAPI ≤ 1 predicted MTX-R independent of the FIGO score (hazard ratio 2.9, *P* = 0.04), with an absolute risk of MTX-R in women with a UAPI ≤ 1 of 67% (95% CI 53–79%) compared with 42% (95% CI 24–61%) with a UAPI > 1 (*P* = 0.036).

Doppler ultrasound also has a potential for following disease response to chemotherapy. Cystic vascular spaces in the myometrial mass of invasive disease show regression during successful chemotherapy. These changes follow decline in serum *β*-hCG levels [2, 6].

**Figure 5:**
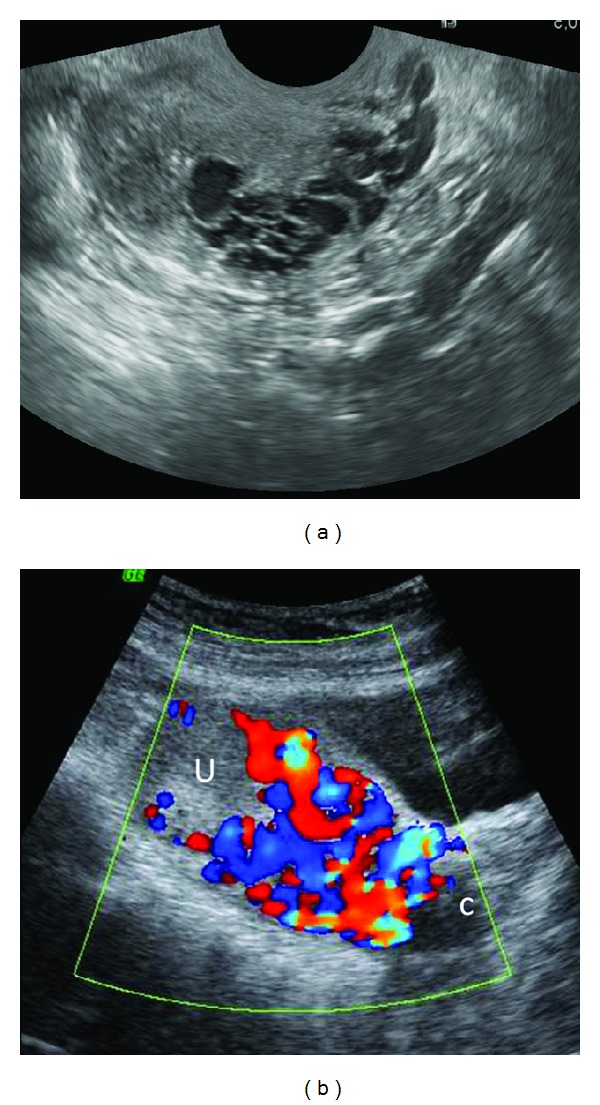
Uterine vascular malformation presenting with recurrent vaginal bleeding following successful treatment of invasive mole. Sagittal TVS image (a) of the uterus reveals multiple tortuous, serpiginous anechoic spaces in the myometrium. Color Doppler TAS (b) reveals a mosaic pattern of color signal within the spaces. U = uterus; C = cervix. No evidence of any myometrial mass is seen to suggest a residual GTN.

**Figure 6:**
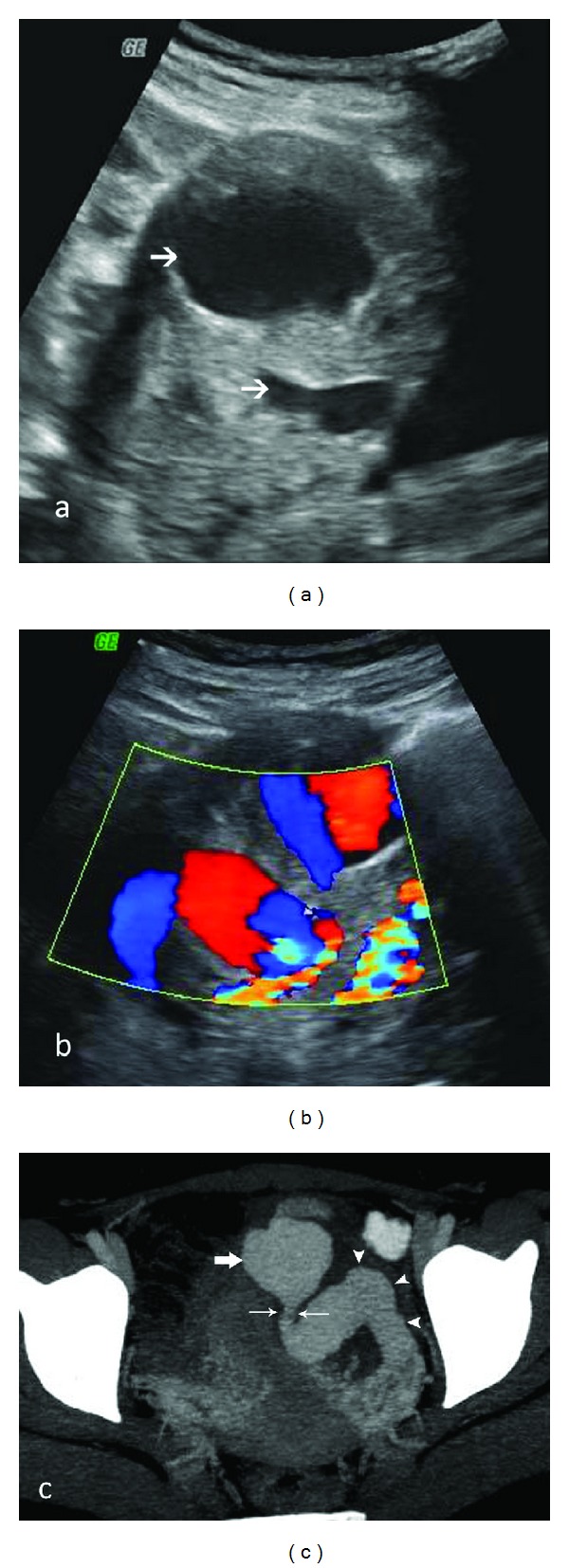
A 25-year-old patient presenting with episodic torrential vaginal bleeding, 3 months after completion of chemotherapy for choriocarcinoma. *β*-hCG levels were not raised. Grey-scale TAS (a) shows two large anechoic lesions in the myometrium (arrows). Color Doppler (b) demonstrates a whirled to-and-fro color flow in these cystic spaces. CT angiogram image (c) reveals a grossly dilated left uterine artery (arrowheads) with a saccular pseudoaneurysm (thick arrow) showing a narrow neck (thin arrows) arising from it and protruding into the adjoining myometrium.

GTN is the commonest cause of uterine vascular malformations (arterio-venous shunts and pseudoaneurysms) and Doppler ultrasound can be a useful tool for demonstrating them (Figures 5 and 6) [2, 6, 19]. These vascular abnormalities may persist in up to 15% of cases following complete response to chemotherapy as a result of residual scarring. These are not considered to be significant if asymptomatic and associated with normal serum and urinary *β*-hCG levels. The abnormalities may regress spontaneously on follow-up imaging. However, rising *β*-hCG levels should prompt search for a recurrent GTN with whole-body imaging [2].

### 4.2. Computed Tomography (CT)

**Figure 7:**
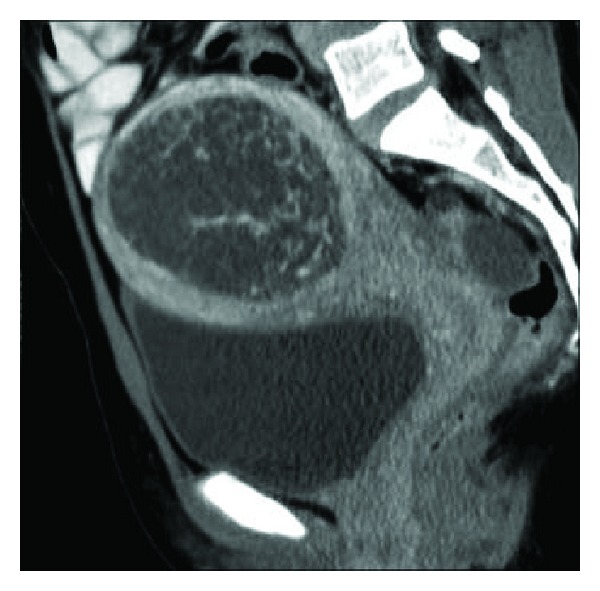
Sagittal reformation of contrast enhanced CT in a patient with complete hydatiform mole shows a large low attenuation, central uterine mass with intact surrounding myometrium.

**Figure 8:**
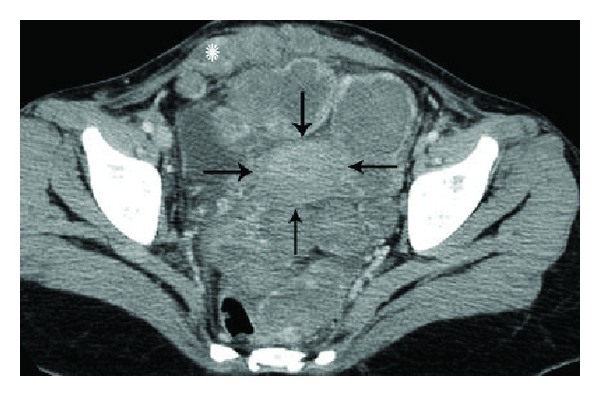
Choriocarcinoma in a 23-year-old patient who presented 2 years after a normal pregnancy with abdominal distension and markedly raised *β*-hCG levels. Axial contrast enhanced CT image of the pelvis reveals extensive, heterogeneously enhancing extrauterine tumor deposits in the pelvis involving pouch of Douglas, left parametrium, anterior peritoneum, and both recti (asterisk). Uterus is marked with black arrows.

CT is principally used for detection of metastatic disease (discussed in a later section). Uterine disease is seen as an enlarged uterus with focal irregular low attenuation lesions (Figure 7) on CT pelvis, which may also demonstrate bilateral ovarian enlargement with multiple theca lutein cysts. Parametrial disease spread is seen as enhancing tissue in this region (Figure 8) [2]. CT can also identify vascular malformations resulting from GTN (Figure 6(c)) [20].

### 4.3. Magnetic Resonance Imaging (MRI)

**Figure 9:**
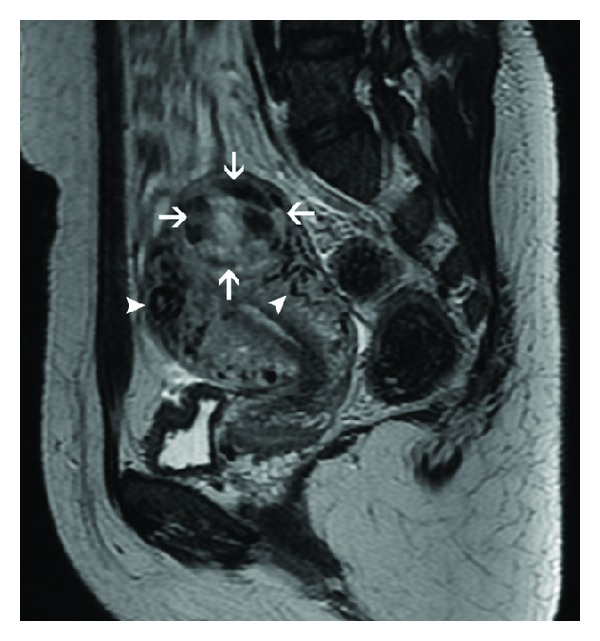
Sagittal T2-weighted MR image in a patient with invasive mole demonstrates a heterogeneous, hyperintense uterine mass in the fundus with a myometrial epicenter (arrows). Tortuous flow voids (arrowheads) consistent with vessels are seen in the adjoining myometrium, indicative of tumor hypervascularity.

**Figure 10:**
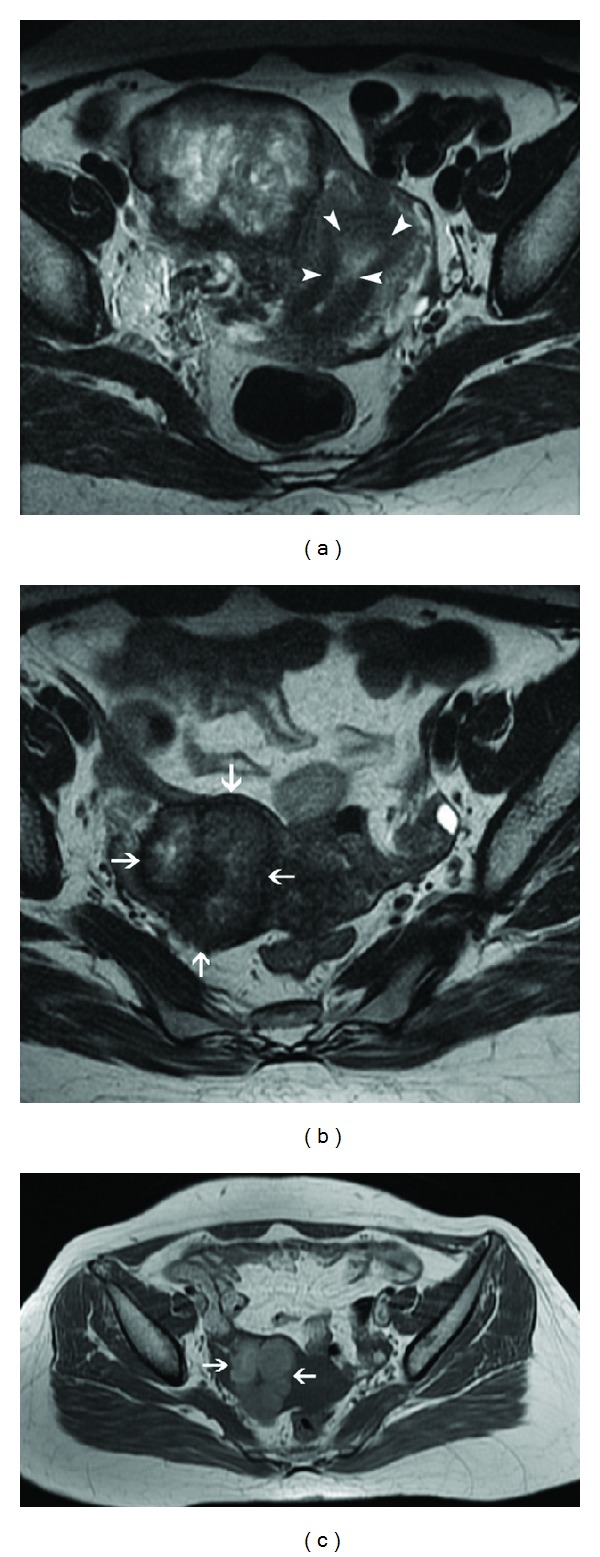
Choriocarcinoma in a 22-year-old patient with extremely raised *β*-hCG levels, 3 months after an abortion. Axial T2-weighted MR image (a) reveals a heterogeneous, hyperintense mass in the right lateral myometrium with parametrial extension. Note is made of a relatively preserved endometrial cavity (arrowheads). Postchemotherapy follow-up MRI ((b), (c)) demonstrates interval regression of the trophoblastic tumor (arrows) with reduction in its size, heterogeneity, and signal hyperintensity on T2-weighted image (b). Hyperintense signal of the lesion (arrows) on T1-weighted image (c) indicates intralesional hemorrhage, a known occurrence following chemotherapy.

MRI does not have a role in routine assessment of pelvic disease. It is sometimes used as a problem-solving tool to assess the depth of myometrial invasion and extrauterine disease spread in equivocal and complicated cases, suspected PSTT, and recurrent GTN [1, 2]. MRI appearance of molar pregnancy can be relatively nonspecific and difficult to distinguish from retained products of conception or ectopic pregnancy [21, 22]. First trimester MRI may reveal little or no abnormality. In the second trimester, hydatiform mole is visualized as a heterogeneously hyperintense tumor distorting the normal zonal architecture on T2-weighted images which may show numerous characteristic cystic spaces. It causes uterine enlargement and distension of the endometrial cavity with indistinct boundary between the endometrium and myometrium. On T1-weighted images, it is isointense or mildly hyperintense to the myometrium with areas of hemorrhage, seen as focal signal hyperintensity [23, 24]. Diffuse myometrial involvement by the tumor is seen as diffuse myometrial signal hyperintensity with obliteration of the normal zonal anatomy. Invasive GTN has similar signal characteristics however with a myometrial epicenter (Figure 9), invasion into parametrium (Figure 10), and more frequent hemorrhage and necrosis [23]. MRI is superior to ultrasound for identification of parametrial invasion, which is seen as heterogeneous T2 hyperintense masses beyond the confines of the uterus (Figure 10) [2, 25].

On contrast enhanced dynamic MRI, viable trophoblastic tissue with surrounding inflammatory response is seen as marked enhancement on early arterial phase [26]. Tumor hypervascularity is also seen as prominent tortuous flow voids in the tumor and adjoining myometrium, parametrium, and adnexae on both T1 and T2-weighted images with engorgement of internal iliac and arcuate vessels with respect to the external iliac artery (Figure 9) [24]. However, the hypovascular form of PSTT has been demonstrated to be hyperintense to myometrium on both T1- and T2-weighted images with absence of prominent vascularity or flow voids and poor enhancement on postcontrast images [2].

With successful treatment, uterine volume and tumor hypervascularity decrease with restoration of normal zonal anatomy on T2-weighted images and reduction of heterogeneous appearance (Figure 10). Intralesional hemorrhage may occur (Figure 10(c)). MRI findings usually normalize at 6–9 months following effective chemotherapy. Persistent uterine vascular malformations are seen as residual tortuous and coiled vessels within a thickened myometrium [2, 20, 24].

**Figure 11:**
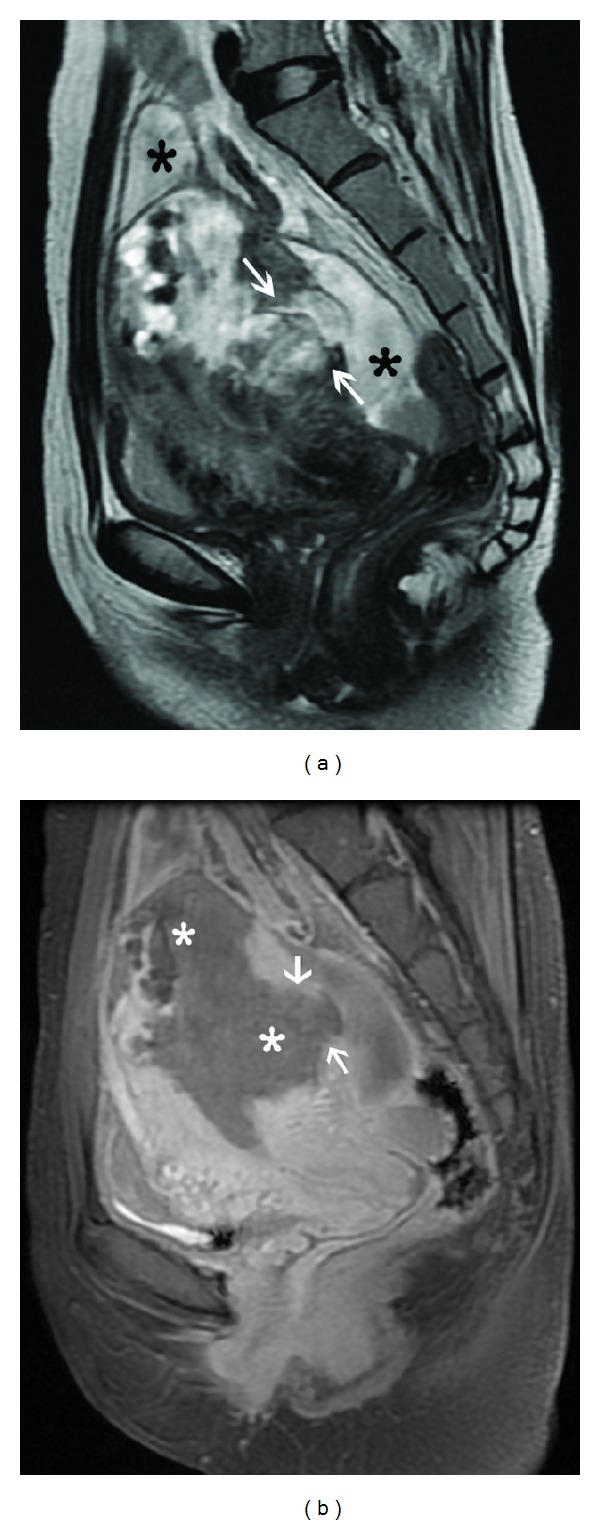
Bulky choriocarcinoma with myometrial rupture. Sagittal T2-weighted image (a) shows an ill-defined heterogeneous mass in the uterine fundus and posterior corpus with full thickness myometrial penetration (arrows) and associated fluid collection (black asterisk) around the uterus. Sagittal contrast-enhanced fat-suppressed T1-weighted MR image (b) demonstrates the mass to be completely necrotic with a large myometrial perforation showing focal continuity (arrows) with the collection in the pouch of Douglas.

Bulky necrotic trophoblastic tissue in the uterine wall may serve as a nidus for infection and cause severe uterine sepsis. If untreated, GTN may penetrate into myometrium with resultant uterine perforation and serious intraperitoneal hemorrhage (Figure 11) [6].

### 4.4. Conventional Angiography

**Figure 12:**
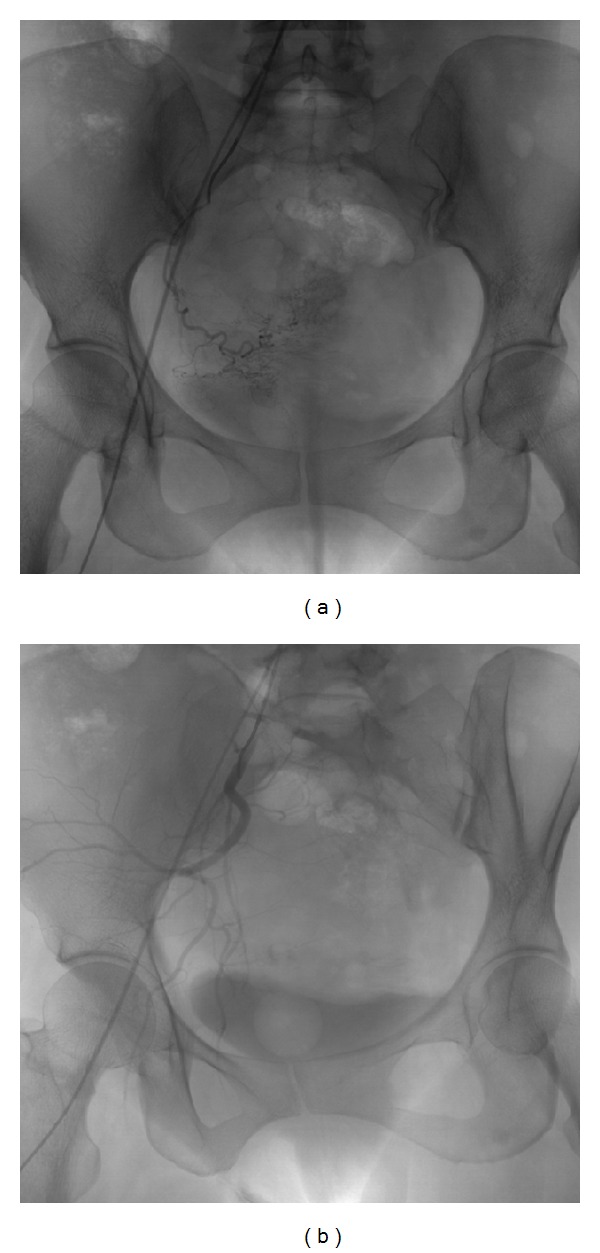
Conventional angiogram (a) in a 23-year-old nulliparous woman with GTN and recurrent massive intraperitoneal bleed showing a uterine vascular malformation on the right side. Selective right uterine artery catheterization was performed via right common iliac artery approach followed by embolization with polyvinyl alcohol gel foam. Postembolization angiogram (b) demonstrates complete obliteration of the vascular malformation.

Although color Doppler ultrasound is the modality of choice for diagnosing uterine vascular malformations, angiography is the preferred method in patients who may potentially undergo embolization for management of the vascular malformations persisting despite complete response to chemotherapy and complicated by refractory, life threatening vaginal, or intraperitoneal hemorrhage (seen in only 2% of cases) [19, 27]. Traditionally, these cases have been managed surgically with uterine artery ligation or hysterectomy. However, selective uterine artery catheterization and embolization are a safe and effective alternative treatment with preservation of fertility in these patients of reproductive age group (Figure 12) [2, 6, 19, 27]. Therapeutic target is 80% or more reduction in the size of the malformation [27]. Repeated embolotherapy may be required for recurrent bleeding [6]. Chances of uterine infarction are low due to extensive pelvic collateralization [2]. Pain requiring opiate analgesia is a frequent complication of treatment [27].

Other indications for conventional angiography in GTN include selective embolization of isolated vaginal metastases and chemoembolization of hepatic metastases. Angiography characteristically demonstrates the hepatic metastasis as a hypervascular mass with aneurysmal dilatation of peripheral ends of hepatic arteries in the arterial phase and persistent vascular lakes in the venous phase [2].

### 4.5. Metastatic Work-Up

**Figure 13:**
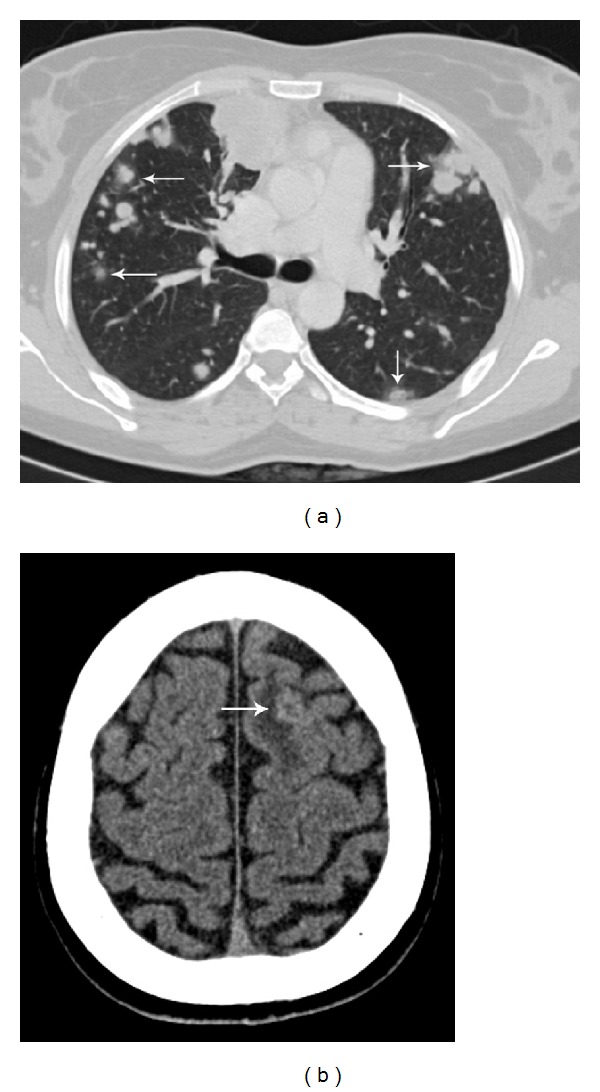
Choriocarcinoma with widespread metastases, presenting with seizures. CT chest (a) shows multiple metastatic lung nodules. Ground glass halo (arrows) around many of these nodules represents hemorrhage. Noncontrast CT brain (b) demonstrates a round, mildly hyperdense lesion (arrow) at gray-white matter junction in the left frontal lobe with surrounding vasogenic edema suggestive of hemorrhagic brain metastasis.

CT scan is more sensitive than chest radiograph for diagnosing lung metastases. Parenchymal lung metastases are seen as multiple, rounded, soft tissue density lesions, usually up to 3 cm in size. The lesions may be solitary, miliary and may rarely show cavitation. A large amount of trophoblastic tissue embolized into the pulmonary vasculature may result in features of acute pulmonary artery hypertension. CT may demonstrate large intravascular tumor thrombus and resultant lung infarct. Both parenchymal and intravascular metastases may show hemorrhage due to their hypervascular nature, resulting in air space shadowing or ground glass opacity (Figure 13(a)). Less common endobronchial tumors may present with endobronchial obstruction. Pleural effusion may result from bleeding of lung nodules into pleural space [2].

Asymptomatic patients without lung metastases are unlikely to have brain metastases and do not require brain MRI or CT. However, patients with known lung or vaginal metastases are at significant risk of central nervous system involvement and should be screened with MRI or CT to exclude brain metastases [2, 9]. Brain metastases are usually multiple, located at grey-white matter junction, and associated with hemorrhage and surrounding edema. Due to hemorrhage, the lesions appear hyperdense on unenhanced CT (Figure 13(b)) and show variable signal intensity on MRI depending on the age of the intralesional hemorrhage. Owing to their hypervascular nature, the lesions usually enhance well [1, 2, 12].

**Figure 14:**
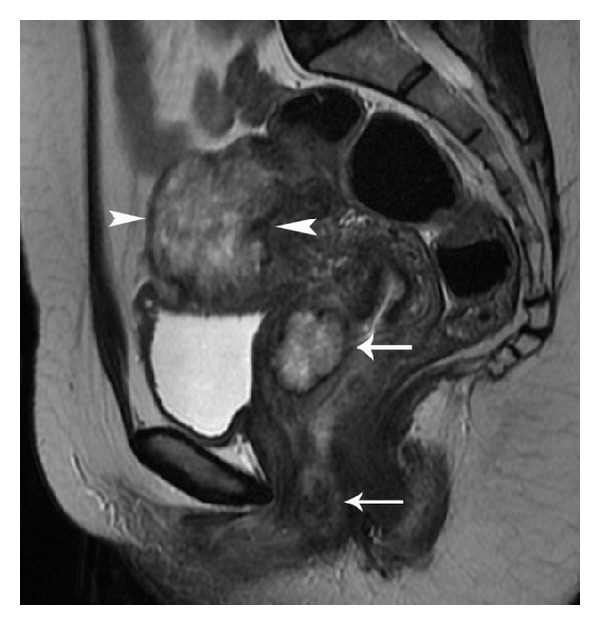
GTN with vaginal metastases. Sagittal T2-weighted MR image shows a lobulated heterogeneously hyperintense myometrial mass with ill-defined margins (arrowheads) and effacement of normal zonal anatomy of uterus. Two morphologically similar lesions (arrows) are seen in the vaginal wall consistent with metastases, which were missed on initial ultrasound.

Vaginal disease usually results from contiguous spread from the uterine lesion. Vaginal metastases are usually evaluated with MRI which demonstrates T2 hyperintense vaginal wall lesion with indistinct margins (Figure 14) [2, 24]. Isolated vaginal metastases may be effectively managed with selective embolization [2].

Liver metastases mark poor prognosis and occur later in the course of disease. Ultrasound screening of the liver may be carried out at the time of pelvic assessment. However, patients with known lung or vaginal metastases or high risk factors should be staged with CT abdomen. Liver metastases are typically seen as multiple hypervascular lesions on both CT and MRI, showing avid contrast enhancement in the arterial phase and sometimes hemorrhagic transformation. These are indistinguishable from other hypervascular hepatic tumors on CT and should never be biopsied due to a risk of fatal hemorrhage [2].

Potential role of FDG PET (18-fluoro-deoxy-glucose positron emission tomography) and PET/CT in the evaluation of recurrent or metastatic disease is a topic of active interest. Preliminary studies suggest that FDG PET is potentially useful in providing precise mapping of metastases and tumour extent, monitoring treatment response, and detecting recurrent or residual disease after chemotherapy. It may be used as problem-solving tool in cases with diagnostic ambiguity on conventional imaging techniques and laboratory tests. However, careful evaluation in combination with other imaging modalities needs to be done to reduce the risk of false positive and negative results [28–33].

### 4.6. Staging and Classification of GTN with Impact on Management

Various staging systems and classifications based on morphology, anatomy, and clinical prognostic scoring of GTN are followed across the world. No single system has gained a universal acceptance. Treatment selection criteria are not uniform everywhere [34–45]. In order to address these issues, the International Society for the Study of Trophoblastic diseases, the International Gynecologic Cancer Society, and International Federation of Gynecology and Obstetrics (FIGO) recommended a new FIGO staging (Table 1(a)) and risk scoring system (Table 1(b)) for GTN after much deliberation and discussions. This is comprised of a combination of basic FIGO stages and simplified WHO scoring system which was adopted in 2000 [3, 9].

Chemotherapy is the mainstay of the management of invasive mole and choriocarcinoma due to exquisite chemosensitivity of most of these lesions, especially if detected early and classified correctly [1–3, 6]. It has been suggested that low risk groups (WHO score ≤ 6) should be managed with a single agent chemotherapy (methotrexate or actinomycin-D) to maximize the cure rates while minimizing toxicity [3, 9, 46–48]. The treatment of choice for high-risk group is combination chemotherapy. Currently, the most widely used combination is EMA-CO (etoposide, methotrexate, actinomycin-D, cyclophosphamide, and vincristine) [3, 9, 49, 50]. Appropriate management results in overall high cure rates (100% for low risk and 80–90% for high-risk group) [2, 9].

### 4.7. Posttreatment Follow-Up

After completion of chemotherapy and normalization of *β*-hCG levels, patients are followed up with serial *β*-hCG levels for one year, although follow-up protocols vary at different institutes [2, 9]. Contraception should be maintained during this period to optimize the chance of detecting a relapse, thus allowing prompt treatment, and to allow DNA repair or apoptosis of the developing ova, potentially damaged by the teratogenic effect of chemotherapy [2, 9]. Follow-up imaging after chemotherapy is performed in case of a rise in *β*-hCG levels to differentiate a normal gestation from disease relapse and to diagnose any complications. Vast majority of patients of GTN treated with chemotherapy can anticipate normal reproductive function [2, 9, 51–53]. Subsequent pregnancy should be evaluated with early ultrasound to confirm normality. *β*-hCG levels should be obtained at 6 and 12 weeks after the subsequent pregnancy [2, 9, 52] to exclude occult trophoblastic disease. Also, patients should be warned against possibility of an early menopause and risk of developing second tumors, such as acute myeloid leukemia (reported with etoposide) as an adverse effect of chemotherapy [9].

## 5. Conclusion

Radiologist plays a key role in the initial diagnosis of GTD and guiding disease management and early detection of its complications. Although serum *β*-hCG is a useful biochemical marker for GTD, it is not diagnostic when considered in isolation. Ultrasound is the first line radiological investigation in confirming the diagnosis of GTD in a case suspected on the basis of clinical findings and *β*-hCG levels. Ultrasound in combination with Doppler is also a useful tool for diagnosing invasive disease, assessing treatment response, and detecting local recurrence. MRI is invaluable to assess extrauterine disease spread and complications. Chest radiograph, brain MRI, and body CT are primarily used to rule out metastatic disease. Conventional angiography can be used to manage patients with heavy bleeding or for chemoembolization of hepatic metastases. PET/CT is fast emerging as a promising tool for disease mapping, monitoring treatment response, and identifying recurrent or residual disease after chemotherapy. Prudent use of these imaging techniques permits early diagnosis and appropriate management, contributing to excellent cure rates of the disease.

## Figures and Tables

**(a):** 

Stage I	Disease confined to the uterus.
Stage II	GTN extends outside the uterus but is limited to the genital structures (adnexa, vagina, and broad ligament).
Stage III	GTN extends to the lungs, with or without known genital tract involvement.
Stage IV	All other metastatic sites.

Source: FIGO Oncology Committee (2003) [10].

**(b):** 

Score	0	1	2	4
Age (years)	<40	≥40	—	—
Antecedent pregnancy	Mole	Abortion	Term	—
Interval months from index pregnancy	<4	4–<7	7–<13	≥13
Pretreatment serum *β*-hCG (iu/mL)	<10^3^	10^3^–<10^4^	10^4^–<10^5^	≥10^5^
Largest tumor size (cm) (including uterus)	<3	3–<5	≥5	—
Site of metastases	Lung	Spleen, kidney	Gastrointestinal	Liver, brain
Number of metastases	—	1–4	5–8	>8
Previous failed chemotherapy	—	—	Single drug	Two or more drugs

Source: FIGO Oncology Committee (2003) [10].
